# *Camk2d* upregulation is associated with postoperative cognitive dysfunction in aged rats: pharmacological inhibition of CaMKII attenuates hippocampal neuroinflammation and neuronal apoptosis

**DOI:** 10.3389/fnagi.2026.1975677

**Published:** 2026-10-12

**Authors:** Kaihua Wei, Shiwen Fan, Yujia Zhao, Xiaobin Cai, Xinlei Qin, Jingwen Zhai, Liping Xie

**Affiliations:** 1 Department of Anesthesiology, The First Affiliated Hospital of Shihezi University, Shihezi, China; 2 School of Medicine, Shihezi University, Shihezi, China

**Keywords:** apoptosis, CaMKIIδ, hippocampus, neuroinflammation, POCD

## Abstract

**Background:**

Postoperative cognitive dysfunction (POCD) is a common complication in elderly patients after major surgery. It delays postoperative recovery and increases the risk of neurodegenerative disorders. CaMKIIδ is a key calcium-signaling regulator of neuronal survival and neuroinflammation, yet its functional role in POCD remains poorly understood. This study aimed to identify injury-inducible calcium-signaling genes and to characterize the association of *Camk2d*, the gene encoding CaMKIIδ, with POCD progression in aged rats.

**Methods:**

A POCD model was established in aged rats by sevoflurane anesthesia combined with splenectomy. Cognitive function was evaluated with the Morris water maze (MWM) and Y-maze tests. Hippocampal transcriptome profiling was performed using RNA sequencing and weighted gene co-expression network analysis (WGCNA). Key molecular and pathological alterations were validated by RT-qPCR, Western blotting, ELISA, and histological staining, and the pan-CaMKII inhibitor KN-93 was administered for functional validation. Serum inflammatory cytokines were measured in parallel to characterize peripheral-to-central inflammatory coupling.

**Results:**

Cognitive performance was worst on postoperative day 3, which was therefore identified as the critical injury window of POCD. Hippocampal CaMKIIδ, phosphorylated NF-κB (p-NF-κB), pro-inflammatory cytokines (IL-1β, IL-6, and TNF-α), and pro-apoptotic Bax were markedly upregulated, whereas anti-apoptotic Bcl-2 reached its lowest level and neuronal apoptosis peaked on day 3. KN-93 treatment downregulated CaMKIIδ expression, suppressed neuroinflammation and neuronal apoptosis, and effectively rescued cognitive impairment in POCD rats.

**Conclusion:**

*Camk2d* upregulation is closely associated with POCD pathogenesis, paralleling hippocampal neuroinflammation and neuronal apoptosis. Pharmacological inhibition of CaMKII by KN-93 alleviates POCD, supporting CaMKII signaling as a potential therapeutic target for perioperative cognitive protection.

## 1 Introduction

With the accelerating aging of the global population, the number of elderly patients requiring general anesthesia and major surgery continues to rise. Postoperative cognitive dysfunction (POCD), a common central nervous system complication of surgery, has attracted considerable attention in clinical anesthesiology and neuroscience (Suraarunsumrit et al., 2024; Blokzijl et al., 2021). POCD is primarily characterized by attention deficits, executive dysfunction, and impaired spatial learning and memory (Vu and Smith, 2022). These impairments delay enhanced recovery after surgery, prolong hospitalization, and increase healthcare costs (Zhao et al., 2024; Yang et al., 2022). Of greater concern, longitudinal epidemiological studies have linked POCD to an increased risk of irreversible neurodegenerative diseases such as Alzheimer's disease (Chen et al., 2023; Kapila et al., 2014). Yet no targeted preventive strategy or effective drug is currently available, largely because the pathophysiology of POCD is complex and incompletely understood (Peng et al., 2023; Zhao et al., 2024). Elucidating its molecular pathogenesis and identifying key therapeutic targets are therefore essential.

The hippocampus is a key brain region for spatial information encoding and memory consolidation. Among its subregions, CA1 is particularly vulnerable to ischemic and inflammatory injury, and disruption of its microenvironmental homeostasis is considered a central event in the pathological progression of POCD (Milani et al., 1998). Surgical trauma activates peripheral immune responses, which can transmit stress signals to the central nervous system through blood-brain barrier disruption or vagal afferent pathways (Li et al., 2024; Yang et al., 2020; Fricke et al., 2021), thereby triggering a marked neuroinflammatory response in the hippocampus (Cao et al., 2010; Yang et al., 2020). During this process, aberrantly activated microglia, the resident immune cells of the hippocampus, markedly upregulate pro-inflammatory cytokines such as interleukin-1β (IL-1β), interleukin-6 (IL-6), and tumor necrosis factor-α (TNF-α) (Cibelli et al., 2010; Koh and Joo, 2025; Hu et al., 2018). The persistent inflammatory microenvironment, together with oxidative stress, further disrupts the balance between pro- and anti-apoptotic Bcl-2 family proteins in hippocampal neurons, ultimately leading to neuronal apoptosis and synaptic loss (Mao et al., 2021; Zhang et al., 2020; Liang et al., 2023). Neuroinflammation and neuronal apoptosis are thus recognized as hallmark pathological features of POCD (Yang et al., 2020; Peng et al., 2023). However, these cellular changes are downstream phenotypes driven by dysregulated signaling molecules and genes. Interventions targeting a single inflammatory factor or apoptotic pathway have shown limited efficacy in clinical translation. Identifying the key upstream genes that sense surgical stress and drive these phenotypes is therefore critical for deciphering the molecular mechanisms of POCD.

Under physiological conditions, tightly regulated intracellular calcium signaling is fundamental to hippocampal synaptic plasticity and memory consolidation (Baker et al., 2013; Teyler et al., 1994; Raymond and Redman, 2006). Severe surgical trauma and anesthetic stress, however, can disrupt neuronal calcium homeostasis and cause intracellular calcium overload (Wei, 2011; Zhang et al., 2022). CaMKIIδ is a key sensor of aberrant intracellular calcium signaling. Upon overexpression and activation, it promotes the dissociation of NF-κB from its inhibitory complex and its subsequent nuclear translocation, initiating a broad transcriptional inflammatory cascade; it also serves as an upstream trigger of apoptotic signaling. Indeed, CaMKIIδ dysregulation has been implicated in cerebral ischemia, traumatic brain injury, and neurodegenerative diseases, in which it mediates neuronal injury through inflammatory and apoptotic mechanisms (Xu et al., 2016; Pan et al., 2014; Khodadadi et al., 2025). Whether CaMKIIδ participates in the pathogenesis of POCD, however, remains unknown.

In this study, a POCD model was established in aged rats by sevoflurane anesthesia combined with splenectomy (Lin et al., 2022; Xiao et al., 2023; Wang et al., 2021). Cognitive function was assessed using the Morris water maze (MWM) and Y-maze tests, and hippocampal tissues were subjected to transcriptomic profiling. By integrating differentially expressed genes across postoperative time points with the trajectory of cognitive decline, we identified *Camk2d*, the gene encoding CaMKIIδ, as a key gene that was significantly and persistently upregulated during the acute phase of cognitive impairment. This study evaluated hippocampus-dependent spatial learning and memory using the Morris water maze and Y-maze tests. Mechanistically, the CaMKIIδ–NF-κB signaling pathway was examined in the hippocampal CA1 region.

We then performed validation experiments focusing on inflammation and apoptosis. Specifically, we measured the inflammatory cytokines IL-1β, IL-6, and TNF-α, the pro-apoptotic protein Bax, and the anti-apoptotic protein Bcl-2 in hippocampal tissues, and combined these measurements with TUNEL staining to assess the association of CaMKIIδ with hippocampal neuroinflammation and neuronal apoptosis. To further define the regulatory role of CaMKIIδ in POCD progression, the CaMKII inhibitor KN-93 was administered as a pharmacological intervention (Wang et al., 2021; Hidalgo et al., 2013; Karnan et al., 2023). Inhibition of CaMKII alleviated hippocampal pathological injury, suppressed neuroinflammation and apoptosis, and improved postoperative cognitive function. These findings identify CaMKIIδ as a candidate target for perioperative neuroprotection in elderly patients.

## 2 Materials and methods

### 2.1 Experimental animals and grouping

Fifty-six male Sprague-Dawley rats (16–18 months old, 550 ± 50 g) were purchased from Sipeifu (Beijing) Biotechnology Co., Ltd. (Beijing, China; animal production license No. SCXK (Jing) 2024-0001). Rats were housed in the specific-pathogen-free (SPF) facility of Shihezi University at 23 °C–25 °C and 50%–75% relative humidity under a 12-h light/dark cycle, with free access to food and water. All experimental procedures were approved by the Animal Ethics Committee of Shihezi University (approval No. A2024-227).

The experiment consisted of two stages. In the molecular screening stage, rats were randomly allocated to four groups (*n* = 8 per group): normal control (Ctrl), postoperative day 3 (POD3), postoperative day 5 (POD5), and postoperative day 7 (POD7). In the pharmacological intervention stage, rats were randomly allocated to three groups (n = 8 per group): normal control (Ctrl), POCD model (POCD), and POCD + KN-93.

### 2.2 Model preparation and pharmacological treatment

Control rats received no surgical intervention. All other rats underwent splenectomy under sevoflurane anesthesia delivered via face mask inhalation. The surgical area was shaved and disinfected, and splenectomy was performed under strict aseptic conditions. The incision was then closed in layers and disinfected again. Rats in the POD3, POD5, and POD7 groups underwent behavioral testing at the corresponding postoperative time points, and behavioral testing in the pharmacological intervention stage was performed on POD3. Rats in the POCD + KN-93 group received intraperitoneal KN-93 at 5 mg·kg^−1^·d^−1^ for 7 consecutive days, beginning 6 days before surgery and ending on the day of surgery. Tissue collection in the Ctrl group was performed when the final batch of animals completed the experiment. The KN-93 dosage (5 mg·kg^−1^·d^−1^, i.p.) used in this study was adopted from previous rat studies. This dose effectively suppresses CaMKII activity in vivo with negligible systemic toxicity (Yousif et al., 2003).

### 2.3 Morris water maze test

The Morris water maze (MWM) apparatus consisted of a black circular pool (150 cm in diameter, 60 cm in height) filled with water maintained at 22 ± 1 °C to a depth of 35 cm, which prevented the rats from touching the pool bottom. The pool was virtually divided into four quadrants. A hidden circular platform (10 cm in diameter) was placed at the center of one quadrant and submerged 1–2 cm below the water surface. The testing environment was kept quiet with uniform, dim lighting. Acquisition training was performed for 5 consecutive days before surgery, with four trials per day and a maximum duration of 60 s per trial. The spatial probe test was then performed at the corresponding postoperative time points, and behavioral parameters were recorded over a 60-s period. All movement trajectories and data were automatically analyzed with Tracking Master V5.0 software.

### 2.4 Y-maze test

A standard Y-maze apparatus was used for the spontaneous alternation test. The three arms (50 cm long, 10 cm wide, and 30 cm high) were arranged at 120° angles and labeled A, B, and C. The apparatus was connected to Tracking Master V5.0 software for automatic recording of behavioral data. One day before the formal test, rats were placed at the center of the maze and allowed to freely explore the three arms for habituation (10 min per session). During the formal test, free exploration was recorded over a 5-min period. The total number of arm entries and the number of effective alternations were extracted. The spontaneous alternation rate was calculated as follows: spontaneous alternation rate = actual alternations/(total arm entries − 2) × 100%.

### 2.5 H&E staining

Hippocampal tissues were fixed and embedded in paraffin, and sections were cut at a thickness of 5 μm. Deparaffinization was performed through sequential immersion in xylene I, II, and III (15 min each), followed by absolute ethanol I and II (5 min each), 95% ethanol (5 min), and 85% ethanol (5 min). Staining was then carried out with a hematoxylin and eosin (H&E) staining kit (Beijing Solarbio Science & Technology Co., Ltd., Beijing, China; catalog No. G1120) according to the manufacturer's instructions. After dehydration and mounting, pathological images were captured with an Olympus BX53 light microscope (Olympus, Tokyo, Japan).

### 2.6 Nissl staining

Paraffin sections were deparaffinized and rehydrated following the same procedure as for H&E staining. The sections were then stained with a toluidine blue Nissl staining kit (Beijing Solarbio Science & Technology Co., Ltd.; catalog No. G1436) according to the manufacturer's instructions. After staining, the sections were air-dried, cleared in xylene for 5 min, and mounted with neutral balsam. Tissue morphology was observed and imaged with an Olympus BX53 light microscope.

### 2.7 TUNEL staining

Hippocampal paraffin sections were routinely deparaffinized and rehydrated. Proteinase K was applied for permeabilization at 37 °C for 20 min, followed by PBS washing. Equilibration buffer was added and incubated at room temperature for 10 min. TUNEL reaction labeling solution was then applied and incubated at 37 °C for 1 h in the dark, followed by PBS washing. Nuclei were counterstained with DAPI for 8 min in the dark, and sections were mounted with antifade mounting medium. Apoptotic cells were observed and imaged with an Olympus FV3000 laser confocal microscope (Olympus, Tokyo, Japan). The ratio of apoptotic cells to total cells was calculated.

### 2.8 ELISA

Rat-specific ELISA kits for IL-1β, IL-6, and TNF-α (Shanghai Mlbio Science & Technology Co., Ltd., Shanghai, China; catalog Nos. ml037361, ml106838, and ml002859) were used to measure the corresponding inflammatory cytokine levels in hippocampal tissues. Assays were performed according to the manufacturer's instructions. After color development, absorbance was measured at 450 nm with a microplate reader (BioTek, USA), and the concentrations of target proteins were calculated. Serum IL‑1β, IL‑6 and TNF‑α were detected using the same batch of ELISA kits, with results expressed in pg/mL. For hippocampal samples, cytokine levels were normalized to the total protein of tissue homogenate and presented as pg/mg protein.

### 2.9 Western blot

Total protein was extracted from hippocampal tissues with RIPA lysis buffer containing protease and phosphatase inhibitors, and protein concentration was determined with a BCA protein assay kit (Beyotime Biotechnology, Shanghai, China). Equal amounts of protein were separated by SDS-PAGE and transferred onto PVDF membranes (Merck Millipore, USA). The membranes were blocked with 5% non-fat milk at room temperature for 1 h and then incubated with primary antibodies at 4 °C overnight: CaMKIIδ (Proteintech, Wuhan, China), Bax (Proteintech, Wuhan, China), Bcl-2 (Proteintech, Wuhan, China), NF-κB (Proteintech, Wuhan, China), p-NF-κB (Proteintech, Wuhan, China), and GAPDH (Proteintech, Wuhan, China). The next day, the membranes were washed with Tris-buffered saline containing Tween 20 (TBST) and incubated with HRP-conjugated secondary antibodies (Proteintech, Wuhan, China) at room temperature for 1 h. Signals were developed with a chemiluminescence reagent, and images were captured with a ChemiDoc imaging system (Bio-Rad, USA). Band intensities were quantified by densitometry with ImageJ software, with GAPDH as the internal reference.

### 2.10 RT-qPCR

Total RNA was extracted from hippocampal tissues with the TransZol Up kit (TransGen, Beijing, China; catalog No. ET111), and RNA concentration and purity were assessed with a NanoDrop spectrophotometer (Thermo Fisher, USA). One microgram of total RNA was reverse-transcribed into cDNA with the PrimeScript reverse transcription kit (Takara, Japan). Quantitative PCR was performed with PerfectStart Green qPCR Master Mix (TransGen Biotech, Beijing, China) on a CFX96 real-time PCR system (Bio-Rad, USA) under the following conditions: 95 °C for 30 s, followed by 40 cycles of 95 °C for 5 s and 60 °C for 30 s. GAPDH was used as the internal reference, and relative mRNA expression levels of target genes were calculated with the 2^−ΔΔCt method.

### 2.11 RNA-seq

RNA-seq was performed on hippocampal tissues with three biological replicates per group. Total RNA was extracted with the TRIzol method, fragmented, and reverse-transcribed into double-stranded cDNA, and sequencing libraries were constructed with ligated adapters. Paired-end 150-bp (PE150) sequencing was performed on the NovaSeq X Plus platform by Shanghai Majorbio Bio-pharm Technology Co., Ltd. (Shanghai, China). Differentially expressed genes (DEGs) between groups were identified with DESeq2 using the criteria of |log2(fold change)| > 1 and adjusted *p* < 0.05. Gene Ontology (GO) and Kyoto Encyclopedia of Genes and Genomes (KEGG) enrichment analyses were performed with Goatools and SciPy, respectively. Weighted gene co-expression network analysis (WGCNA) was used to identify gene modules significantly associated with cognitive behavioral parameters. We performed over-representation analysis for common DEGs and MEred module genes to examine autophagy-associated signaling. GO autophagy terms (GO:0006914 and all child terms) and the KEGG autophagy pathway (rno04140) were tested, with Fisher’s exact test and Benjamini–Hochberg correction applied. The MEred module eigengene corresponds to the first principal component of standardized gene expression within the module. Pearson and Spearman correlation tests were used to evaluate its relationships with *Camk2d* expression and individual-animal behavioral outcomes.

### 2.12 Statistical analysis

Statistical analysis was performed with GraphPad Prism 8.0 software (GraphPad Software, San Diego, CA, USA). Normality of data distribution was assessed with the Shapiro–Wilk test. For the escape latency during the MWM training phase, two-way repeated-measures ANOVA followed by Bonferroni's post-hoc test was used. For other multi-group comparisons of independent samples (MWM probe test, Y-maze, Western blot, RT-qPCR, ELISA, and TUNEL quantification), one-way ANOVA followed by Tukey's post-hoc test was applied when the data met the assumptions of normality and homogeneity of variance; otherwise, the Kruskal–Wallis test was used. Data are presented as mean ± SD, and *p* < 0.05 was considered statistically significant. Levene’s test was used to check variance homogeneity. The statistical test selected for each dataset is noted in the relevant figure legend. Pearson or Spearman correlation was adopted according to data characteristics.

## 3 Results

### 3.1 Splenectomy under sevoflurane anesthesia induced postoperative cognitive dysfunction in aged rats

To determine the onset and temporal dynamics of POCD after splenectomy under sevoflurane anesthesia, we established the model and conducted behavioral testing according to the experimental protocol (Figure 1A). During the MWM training phase, escape latency gradually decreased across training days in all groups, indicating that all rats successfully acquired the task (Figures 1B,C). Swimming speed did not differ significantly among groups, excluding baseline motor ability as a confounder of the cognitive assessment (Figure 1D). The spatial probe test revealed marked spatial memory impairment in surgical rats compared with the Ctrl group. The latency to reach the platform site was significantly prolonged, peaking at approximately 36 s on POD3, and then gradually shortened with postoperative recovery (Figures 1E,F). The number of platform crossings was significantly reduced, reaching a minimum of approximately 1 on POD3, and gradually increased thereafter (Figure 1G). Time spent in the target quadrant was also significantly reduced, with the most severe impairment on POD3 (approximately 10 s); this parameter gradually improved over time but remained below the control level (Figure 1H).

**Figure 1:**
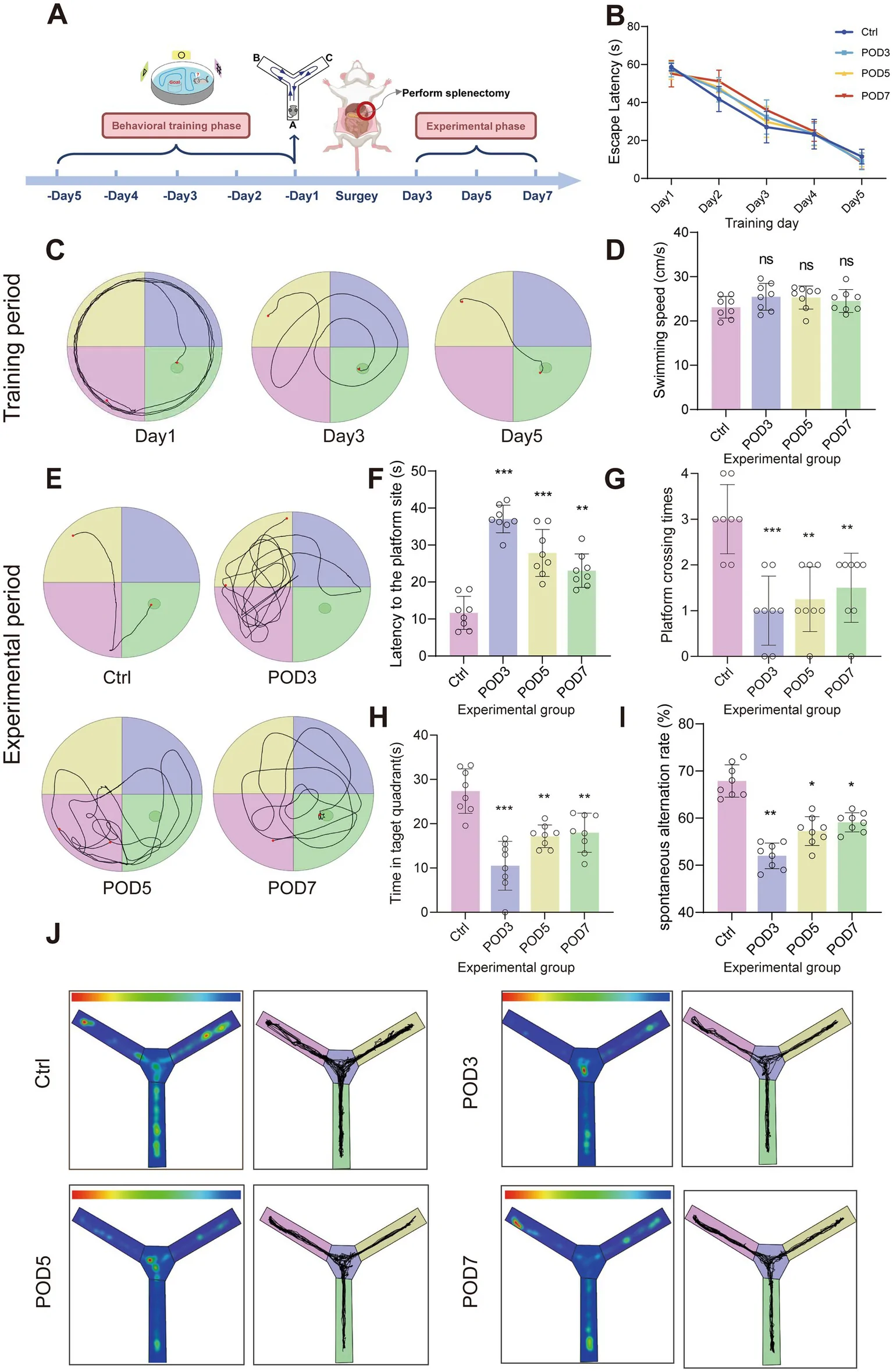
Splenectomy under sevoflurane anesthesia induced cognitive dysfunction in aged rats. **(A)** Schematic of the experimental protocol. Aged rats received 5 consecutive days of MWM training before surgery. On the day after the final training session, rats in the surgery groups underwent splenectomy under sevoflurane anesthesia. MWM probe and Y-maze tests were conducted on postoperative days 3, 5, and 7, with Y-maze habituation performed 1 day before each formal test. **(B)** Escape latency of each group during the training phase (*n* = 8). **(C)** Representative swimming trajectories on training days 1, 3, and 5. **(D)** Swimming speed of each group during the training phase (*n* = 8). **(E)** Representative swimming trajectories of the Ctrl and surgery groups in the probe test. **(F)** Latency to reach the platform site at each postoperative time point (*n* = 8). **(G)** Number of platform crossings at each postoperative time point (*n* = 8). **(H)** Time spent in the target quadrant at each postoperative time point (*n* = 8). **(I)** Spontaneous alternation rate of each group in the Y-maze after surgery (*n* = 8). **(J)** Representative trajectories of the Ctrl and surgery groups in the Y-maze. Data are presented as mean ± SD. Training-phase data were analyzed by two-way repeated-measures ANOVA with Bonferroni's post-hoc test, and other data by one-way ANOVA with Tukey's post-hoc test. *p < 0.05, **p < 0.01, ***p < 0.001 vs. Ctrl group; ns, not significant. MWM, Morris water maze; POD, postoperative day.

The Y-maze spontaneous alternation test further confirmed this dynamic pattern of postoperative cognitive impairment. Compared with the Ctrl group, the spontaneous alternation rate was significantly decreased in all postoperative groups, with the most pronounced reduction on POD3. Although the alternation rate partially recovered over time, it remained significantly lower than the control level (Figures 1I,J). In summary, splenectomy under sevoflurane anesthesia induced marked cognitive dysfunction in aged rats, and POD3 represented the critical time window of POCD.

### 3.2 Postoperative day 3 was the critical window for hippocampal neuronal injury and neuroinflammation in aged rats

To characterize the pathological changes of hippocampal neurons after splenectomy under sevoflurane anesthesia, we first performed H&E staining on the CA1 region. In the Ctrl group, CA1 neurons were arranged in an orderly manner, with distinct nuclei and abundant cytoplasm, and showed no obvious pathological damage. In contrast, neurons in the POD3, POD5, and POD7 groups displayed a disorganized arrangement together with typical injury features, including cell body shrinkage, nuclear pyknosis, and enhanced cytoplasmic eosinophilia. The damage was most severe on POD3. It partially subsided over time but remained evident on POD7 (Figure 2A). Nissl staining further confirmed the postoperative neuronal damage. In the Ctrl group, CA1 neurons contained abundant Nissl bodies with uniform dark blue-purple granular staining. At all postoperative time points, the number of Nissl bodies was markedly reduced and the staining was pale; some neurons showed dissolution or complete loss of Nissl bodies. Again, the POD3 group exhibited the most severe damage (Figure 2B).

**Figure 2:**
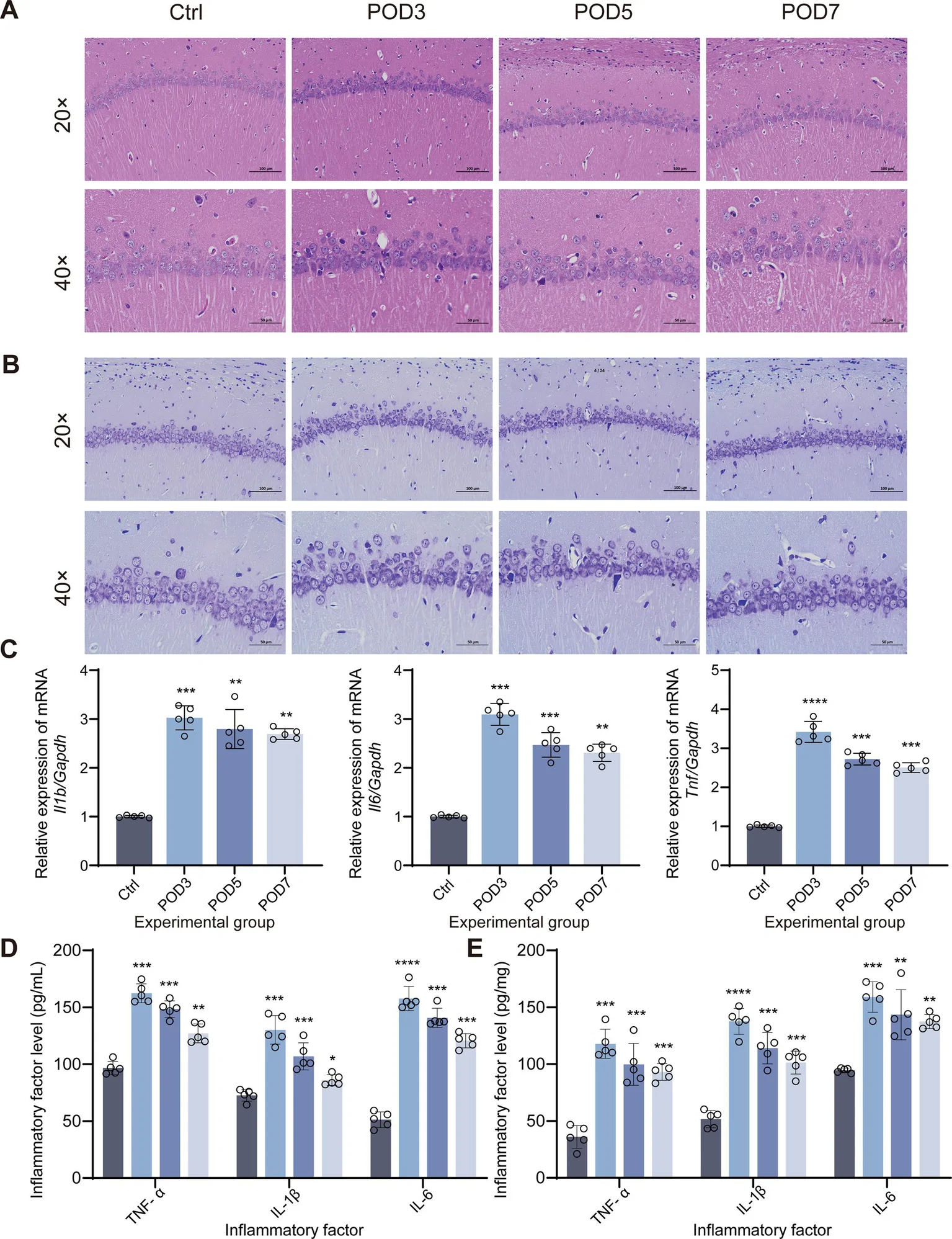
Hippocampal neuronal injury and neuroinflammatory responses in POCD model rats. **(A)** Representative H&E staining images of the hippocampal CA1 region in each group (*n* = 3; scale bars: 100 μm at 20 × and 50 μm at 40×). **(B)** Representative Nissl staining images of the hippocampal CA1 region in each group (*n* = 3; scale bars: 100 μm at 20 × and 50 μm at 40×). **(C)** Relative mRNA expression levels of IL-1β, IL-6, and TNF-α in the hippocampus measured by RT-qPCR (*n* = 5). **(D)** Protein levels of IL-1β, IL-6, and TNF-α in the Serum measured by ELISA (*n* = 5). **(E)** Protein levels of IL-1β, IL-6, and TNF-α in the hippocampus measured by ELISA (*n* = 5). Data are presented as mean ± SD and were analyzed by one-way ANOVA followed by Tukey's post-hoc test. **p < 0.01, ***p < 0.001, ****p < 0.0001 vs. Ctrl group.

We next evaluated the dynamics of postoperative peripheral inflammatory responses by measuring serum IL-1β, IL-6, and TNF-α concentrations via ELISA. Compared with the Ctrl group, serum concentrations of all three cytokines increased significantly after surgery, peaked on POD3, and then gradually declined on POD5 and POD7 (Figure 2D).

We subsequently examined the neuroinflammatory status in the hippocampus at both the transcriptional and translational levels. RT-qPCR results showed that, compared with the Ctrl group, hippocampal mRNA expression of IL-1β, IL-6, and TNF-α was significantly upregulated in all postoperative groups, with all three cytokines peaking on POD3 and gradually declining thereafter (Figure 2C). In line with both the transcriptional trends and the serum temporal profile, ELISA results revealed that hippocampal protein levels of the three cytokines were also highest in the POD3 group. Although they declined to varying degrees at other postoperative time points, they remained significantly higher than the control levels (Figure 2E). These findings indicate that postoperative cognitive dysfunction in aged rats is closely associated with hippocampal neuroinflammation.

The time course of serum cytokines was highly consistent with that of hippocampal cytokines (Supplementary Figures S1A–C). Spearman correlation analysis of paired samples further verified positive correlations between serum and hippocampal cytokine levels. Collectively, these data demonstrate that hippocampal neuroinflammation occurs in parallel with systemic inflammatory activation, supporting crosstalk between peripheral and central inflammatory signals.

### 3.3 Identification of key pathways and co-expression modules by RNA-seq and WGCNA

To identify key genes associated with the onset and progression of POCD, we performed transcriptome sequencing on hippocampal tissues from the Ctrl, POD3, POD5, and POD7 groups. Principal component analysis (PCA) showed tight within-group clustering and clear between-group separation, indicating high reproducibility of the sequencing data (Figure 3A). Intersection analysis identified 691 differentially expressed genes (DEGs) shared by all three postoperative time points relative to the Ctrl group (Figure 3B). KEGG enrichment analysis revealed that these common DEGs were significantly enriched in the axon guidance, calcium signaling, neuroactive ligand–receptor interaction, and apoptosis pathways (Figure 3C).

**Figure 3:**
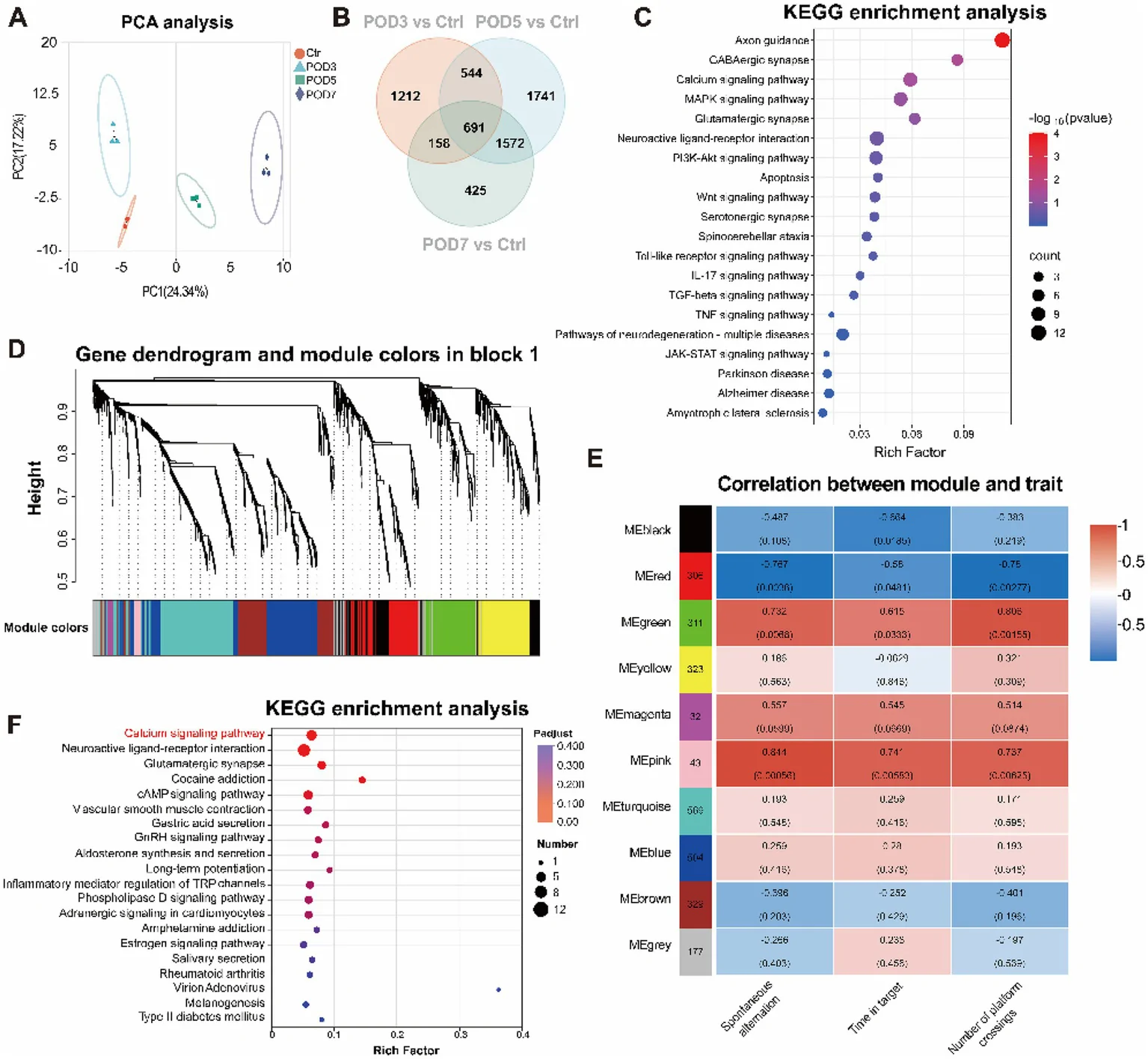
Integrated RNA-seq and WGCNA analysis of the aged rat hippocampus. **(A)** PCA of normalized gene expression data. **(B)** Venn diagram of DEGs from each postoperative time point versus the Ctrl group; the intersection represents the 691 common DEGs. **(C)** KEGG enrichment analysis of the 691 common DEGs, showing the top 20 significantly enriched pathways. Bubble color indicates the adjusted *p* value, bubble size indicates the gene count, and the *x*-axis represents the Rich Factor. **(D)** Gene dendrogram from WGCNA. Genes were hierarchically clustered based on topological overlap, and different colors represent the assigned co-expression modules. **(E)** Module–trait correlation heatmap showing correlations between co-expression modules and behavioral phenotypes. Color represents the correlation coefficient (r), with *p* values in parentheses. **(F)** KEGG enrichment bubble plot of genes in the MEred module. Correlations in **(E)** were assessed using Pearson’s correlation analysis.

To identify core gene modules associated with the cognitive impairment phenotype of POCD, we performed WGCNA using the spontaneous alternation rate, time spent in the target quadrant, and number of platform crossings as phenotypic traits. A total of 10 co-expression modules were identified. Module–trait correlation analysis showed that the MEred module was significantly and negatively correlated with all three cognitive traits (Figures 3D,E); in other words, higher expression of MEred module genes corresponded to poorer cognitive performance. Genes within this module are therefore likely to be closely associated with POCD pathogenesis.

KEGG enrichment analysis of the genes in the MEred module revealed significant enrichment in the calcium signaling and neuroactive ligand–receptor interaction pathways (Figure 3F), consistent with the enrichment profile of the common DEGs. Together, these findings suggest central roles for the axon guidance, calcium signaling, neuroactive ligand–receptor interaction, and apoptosis pathways in the pathogenesis of POCD.

To address the potential involvement of autophagy, we performed over-representation analysis of the common DEGs and the MEred module genes against the GO autophagy terms (GO:0006914 and all descendants) and the KEGG autophagy pathway (rno04140). Contrary to expectation, autophagy-related terms were not significantly enriched in either gene set (fold enrichment 0.15–0.80; all adjusted *p* > 0.7; Supplementary Figure S3), and canonical autophagy genes (*Map1lc3b*, *Sqstm1*, *Becn1*, *Atg5*, *Atg7*, *Atg12*, and *Ulk1*) were not among the common DEGs. At the transcriptional level, autophagy therefore does not emerge as a major coordinated response within the injury-relevant modules, although post-transcriptional or cell-type-specific autophagy dysregulation cannot be excluded.

### 3.4 Identification of *Camk2d* as a core gene associated with POCD

To identify the core genes within the MEred module from a temporal perspective, we performed time-course expression trend clustering on the 306 module genes using the R package Mfuzz. The genes were divided into five clusters. The expression trajectories of Clusters 1, 3, 4, and 5 mirrored the temporal course of cognitive impairment, with expression peaking on POD3, whereas Cluster 2 did not (Figure 4A). KEGG enrichment analysis of these four clusters revealed significant enrichment in the calcium, cAMP, and GnRH signaling pathways, among which the calcium signaling pathway was the most significantly enriched, consistent with the enrichment profile of the MEred module (Figure 4B).

**Figure 4:**
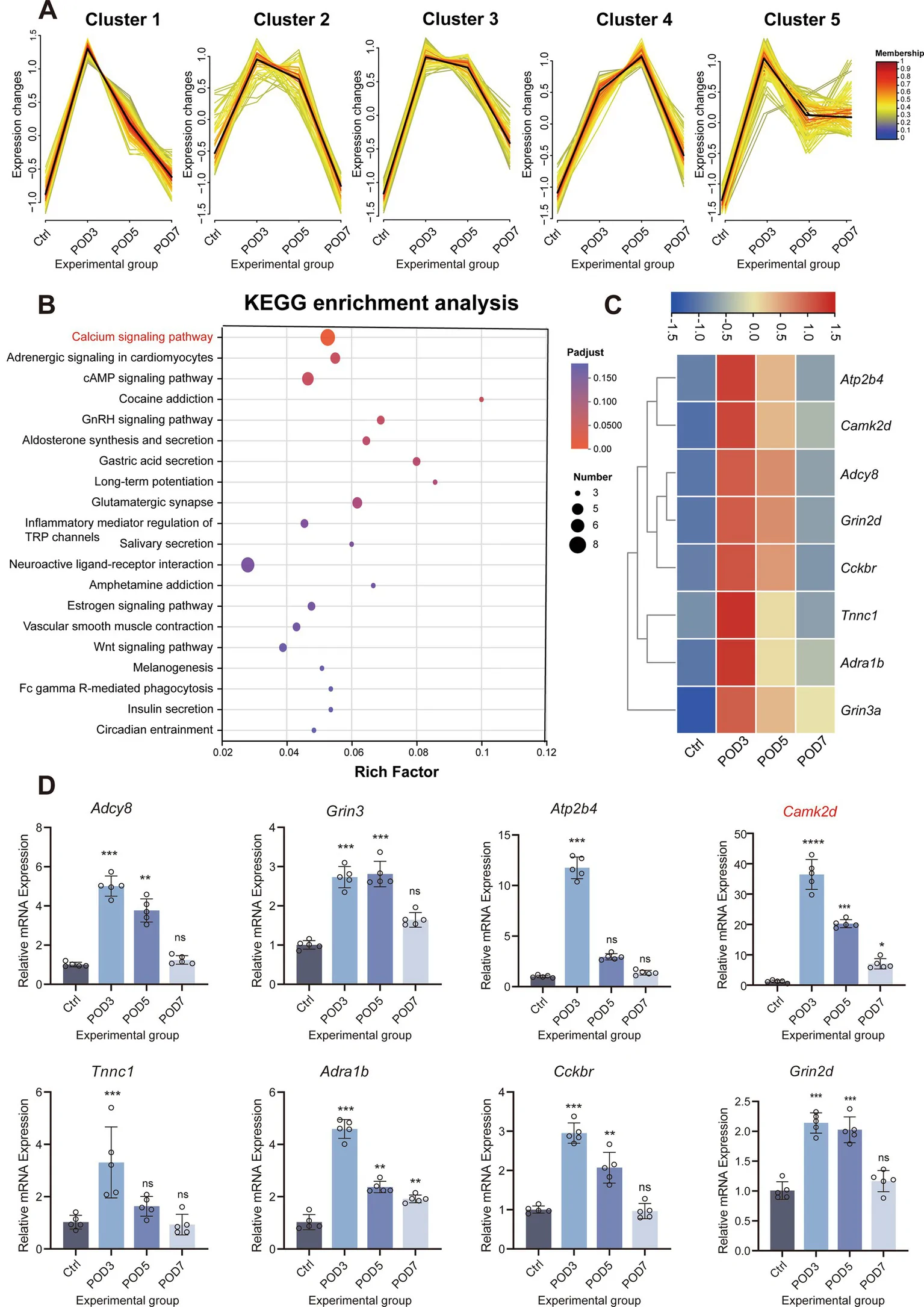
Identification of *Camk2d* as a core gene associated with POCD. **(A)** Time-course expression trend clustering of the 306 genes in the MEred module using Mfuzz, yielding five clusters. Black lines indicate the average expression trajectory of each cluster; colored lines represent individual genes, with color indicating the membership degree of each gene. **(B)** KEGG pathway enrichment analysis of the genes in Clusters 1, 3, 4, and 5, showing the top 20 significantly enriched pathways. **(C)** Heatmap of the eight calcium signaling pathway genes across the Ctrl and postoperative groups. Blue indicates low relative expression and red indicates high relative expression. **(D)** RT-qPCR validation of the mRNA levels of the eight calcium signaling pathway genes (*n* = 5). Data are presented as mean ± SD and were analyzed by one-way ANOVA followed by Tukey's post-hoc test. *p < 0.05, **p < 0.01, ***p < 0.001, ****p < 0.0001 vs. Ctrl group; ns, not significant.

Based on these enrichment results, eight genes in the calcium signaling pathway were extracted from the four clusters, and their expression patterns were visualized in a heatmap. *Atp2b4*, *Camk2d*, *Tnnc1*, and *Adra1b* all peaked on POD3 (Figure 4C). To validate the transcriptomic findings, we measured the mRNA levels of these eight genes by RT-qPCR. The RT-qPCR results were consistent with the RNA-seq trends, and *Atp2b4*, *Camk2d*, *Tnnc1*, and *Adra1b* showed the largest expression changes. Notably, the upregulation of *Camk2d* on POD3 was the most pronounced among the candidate genes. Taken together, these findings suggest that *Camk2d* may serve as a core regulatory gene underlying the onset and progression of POCD.

To examine the relationship between *Camk2d* and the MEred module for individual animals, we calculated the module eigengene for each sample, which represents the first principal component of gene expression within that module. Across all samples, *Camk2d* expression was strongly positively correlated with the MEred module eigengene (Pearson *r* = 0.83, *p* = 7.7 × 10^–4^; Spearman *ρ* = 0.99, *p* = 4.1 × 10^–9^, *n* = 12; Supplementary Figure S4). Both the eigengene and *Camk2d* expression correlated negatively with all three cognitive metrics. For the module eigengene: spontaneous alternation *ρ* = −0.73, *p* = 0.008; time in target quadrant *ρ* = −0.63, *p* = 0.028; platform crossings *ρ* = −0.80, *p* = 0.002. For *Camk2d*: *r* = −0.88, *p* = 2 × 10^–4^; *r* = −0.88, *p* = 1 × 10^4^; *r* = −0.78, *p* = 0.003 (Supplementary Figure S5). These findings confirmed the module–trait association at the individual animal level.

### 3.5 Upregulation of CaMKIIδ was accompanied by increased hippocampal neuroinflammation and apoptosis

To determine the protein expression of CaMKIIδ, the product of the core gene *Camk2d*, and the activation status of hippocampal apoptotic and inflammatory pathways, we performed Western blotting on hippocampal tissues. Compared with the Ctrl group, CaMKIIδ protein expression was significantly elevated in the POD3, POD5, and POD7 groups. It exhibited a time-dependent pattern, peaking on POD3 and gradually declining on POD5 and POD7, consistent with its mRNA trajectory (Figures 5A,B; Figures 4C,D). The pro-apoptotic protein Bax and phosphorylated NF-κB (p-NF-κB), a marker of inflammatory pathway activation, both peaked on POD3 and gradually decreased thereafter. The anti-apoptotic protein Bcl-2 showed the opposite trend: its expression dropped to the lowest level on POD3 and slowly recovered during the postoperative course (Figures 5A,B). RT-qPCR confirmed that the mRNA levels of Bax and Bcl2 changed consistently with their protein levels (Figure 5C).

**Figure 5:**
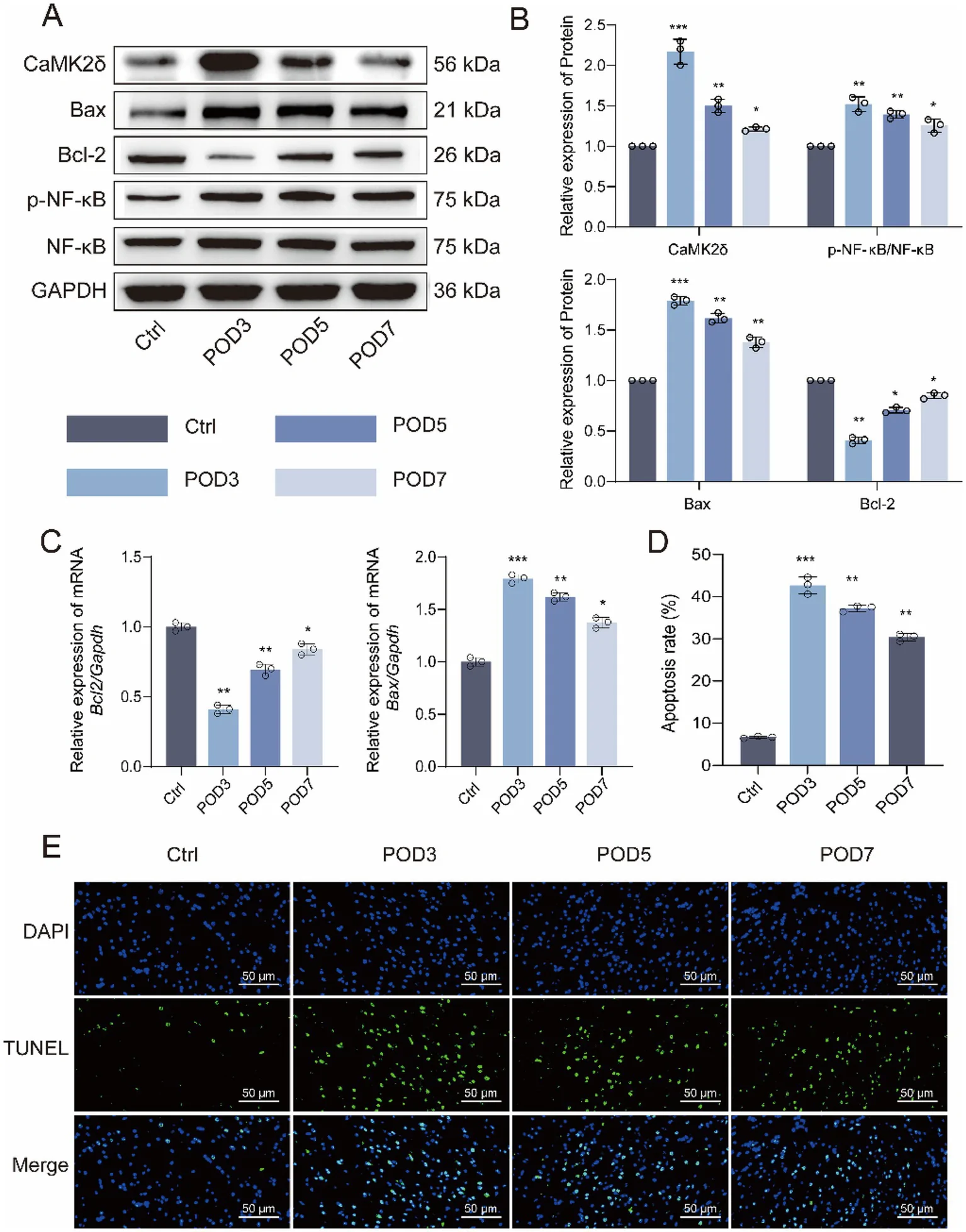
Expression of CaMKIIδ and neuroinflammation- and apoptosis-related proteins in the hippocampus of POCD rats. **(A,B)** Representative Western blot bands and densitometric quantification of CaMKIIδ, p-NF-κB, NF-κB, Bax, and Bcl-2 (*n* = 3). p-NF-κB was normalized to total NF-κB, and the other targets were normalized to GAPDH. (*n* = 3). **(C)** mRNA expression levels of Bax and Bcl2 measured by RT-qPCR (*n* = 3). **(D,E)** Representative TUNEL staining images and quantification of apoptotic cells in the hippocampus (*n* = 3; scale bar: 50 μm). Data are presented as mean ± SD and were analyzed by one-way ANOVA followed by Tukey's post-hoc test. *p < 0.05, **p < 0.01, ***p < 0.001 vs. Ctrl group.

In addition, TUNEL staining confirmed hippocampal apoptosis at the histological level. Compared with the Ctrl group, the percentage of TUNEL-positive cells was significantly increased at all postoperative time points. The apoptosis rate also peaked on POD3 and declined thereafter (Figures 5D,E). These findings indicate that surgery-induced hippocampal apoptosis is markedly time-dependent, with the most severe damage occurring in the early postoperative period followed by gradual alleviation.

### 3.6 Inhibition of CaMKII ameliorated postoperative cognitive dysfunction in aged rats

To determine the functional role of CaMKIIδ in the onset and progression of POCD, the pan-CaMKII inhibitor KN-93 was administered as a pharmacological intervention, and cognitive function was assessed with the MWM and Y-maze tests. During the MWM training phase, escape latency gradually decreased across training days in the Ctrl, POCD, and POCD + KN-93 groups, with no significant difference among groups (Figures 6A,B), indicating that KN-93 itself did not affect learning acquisition. Swimming speed also did not differ among groups (Figure 6D), excluding differences in learning acquisition and baseline motor function as confounders of the cognitive assessment.

**Figure 6:**
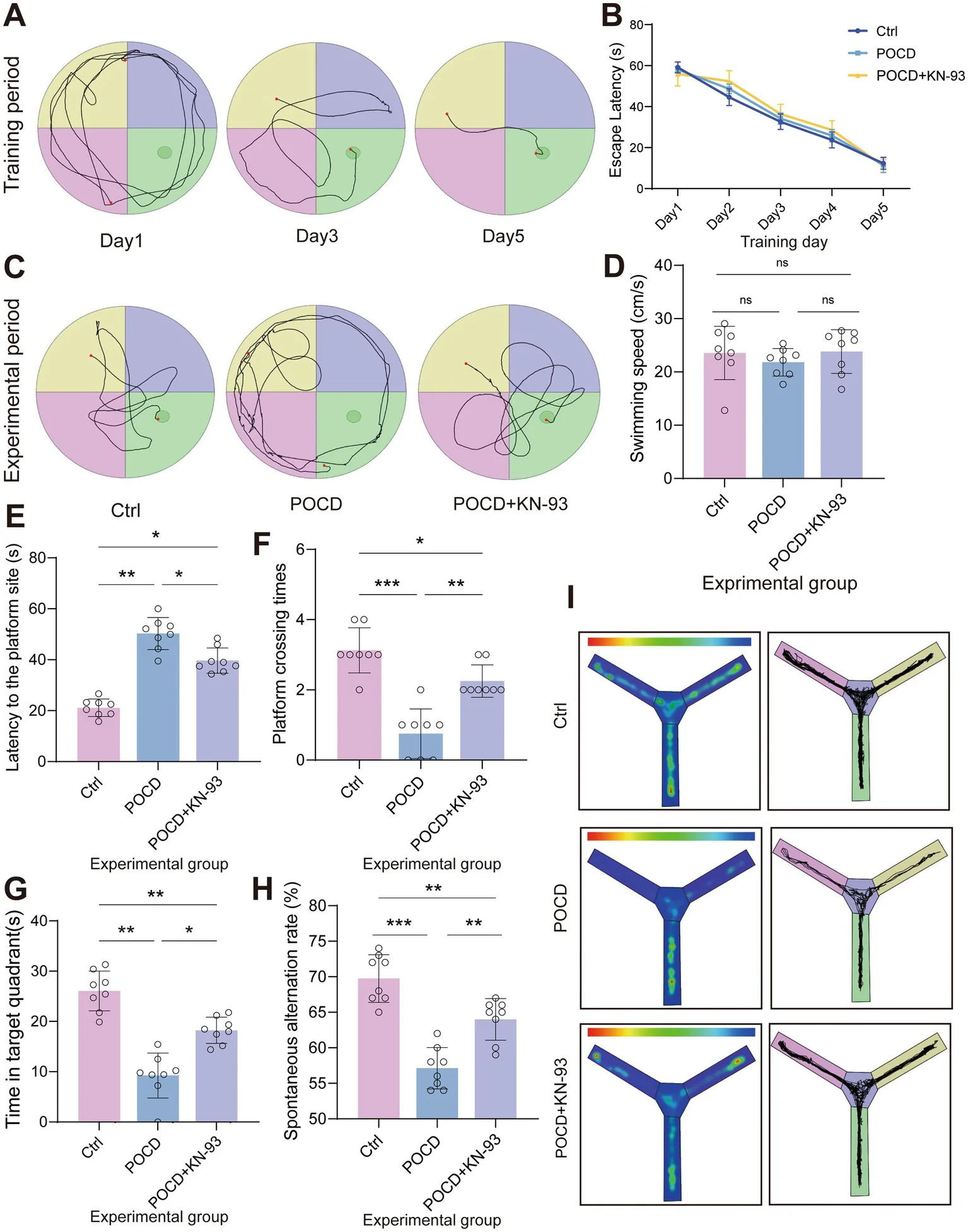
KN-93 ameliorated cognitive dysfunction in aged POCD rats. **(A)** Representative swimming trajectories on training days 1, 3, and 5 of the MWM. **(B)** Escape latency to reach the hidden platform on training days 1–5 in each group (*n* = 8). **(C)** Representative swimming trajectories of each group in the probe test. **(D)** Swimming speed of each group during the training phase (*n* = 8). **(E)** Latency to reach the platform site in the probe test (*n* = 8). **(F)** Number of platform crossings in the probe test (*n* = 8). **(G)** Time spent in the target quadrant in the probe test (*n* = 8). **(H)** Spontaneous alternation rate in the Y-maze (*n* = 8). **(I)** Representative trajectory heatmaps in the Y-maze. Data are presented as mean ± SD. Training-phase data were analyzed by two-way repeated-measures ANOVA with Bonferroni's post-hoc test, and other data by one-way ANOVA with Tukey's post-hoc test. *p < 0.05, **p < 0.01, ***p < 0.001; ns, not significant.

The spatial probe test showed that, compared with the Ctrl group, the POCD group exhibited marked spatial memory impairment. The latency to reach the platform site was significantly prolonged, while the time spent in the target quadrant and the number of platform crossings were both significantly reduced. KN-93 intervention alleviated these impairments: compared with the POCD group, the POCD + KN-93 group showed a significantly shorter latency to the platform site, with the time in the target quadrant and the number of platform crossings both significantly increased (Figures 6C,E–G). The Y-maze spontaneous alternation test yielded consistent results. The spontaneous alternation rate was significantly decreased in the POCD group and significantly improved after KN-93 intervention (Figures 6H,I). In summary, pharmacological inhibition of CaMKIIδ ameliorated spatial memory and working memory impairments in POCD rats.

### 3.7 Pharmacological inhibition of CaMKIIδ attenuated hippocampal neuroinflammation and neuronal apoptosis

To investigate the functional role of CaMKIIδ in POCD, we administered the pan-CaMKII inhibitor KN-93 for in vivo pharmacological intervention. The regulatory effects of CaMKIIδ inhibition on hippocampal neuroinflammation and neuronal apoptosis were assessed by H&E staining, Nissl staining, Western blot, ELISA, and TUNEL staining.

H&E staining revealed that hippocampal CA1 neurons in the Ctrl group were arranged in an orderly manner with intact structure and no evident pathological damage. In the POCD group, neurons displayed disorganized arrangement, apparent reduction in cell number, cell body shrinkage, and degenerative changes. These pathological alterations were markedly attenuated in the POCD + KN-93 group. Nissl staining further corroborated the alleviation of neuronal damage. In the Ctrl group, CA1 neurons contained abundant Nissl bodies with uniform staining. In the POCD group, the number of Nissl bodies was markedly reduced with pale staining, and some neurons exhibited loss of Nissl substance. In the POCD + KN-93 group, the number of Nissl bodies was increased compared with the POCD group, with deeper staining and substantially alleviated neuronal injury (Figure 7A).

**Figure 7:**
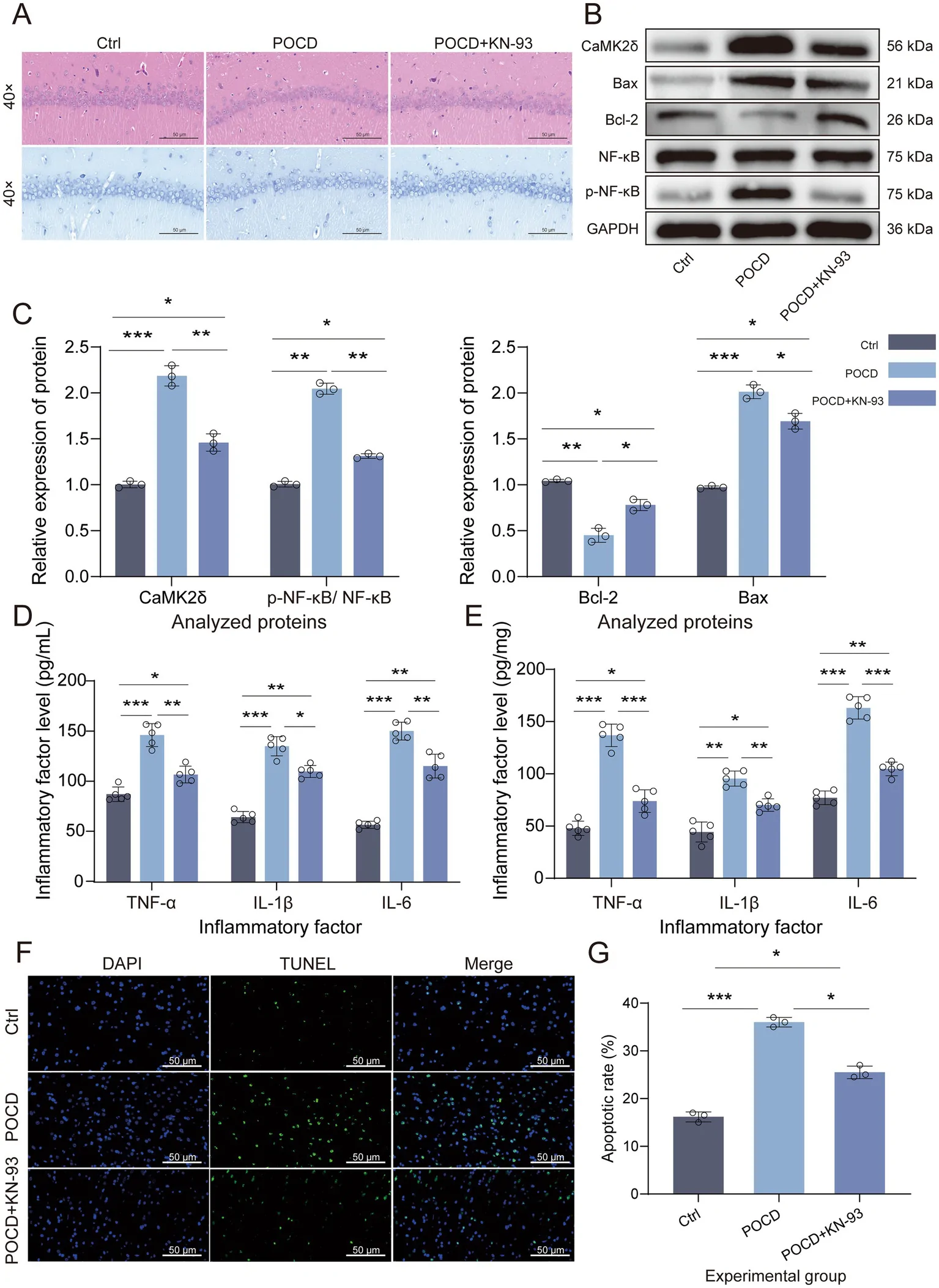
Pharmacological inhibition of CaMKII attenuates hippocampal neuroinflammation and neuronal apoptosis in POCD rats. **(A)** Representative H&E and Nissl staining images of the hippocampal CA1 region (*n* = 3 rats per group, randomly selected; scale bar = 50 μm, 40 × magnification). **(B,C)** Representative Western blot bands and densitometric quantification of CaMKIIδ, p-NF-κB, total NF-κB, Bax, and Bcl-2, with GAPDH as the loading control (*n* = 3 rats per group). The p-NF-κB/NF-κB ratio was calculated. (D) Protein levels of IL-1β, IL-6, and TNF-α in the Serum measured by ELISA (*n* = 5). (E) Protein concentrations of TNF-α, IL-1β, and IL-6 in hippocampal tissues, measured by ELISA (*n* = 5 rats per group). **(F,G)** Representative TUNEL fluorescence images and quantitative analysis of the apoptotic index in the hippocampal CA1 region (*n* = 3 rats per group; scale bar = 50 μm, 40 × magnification). The apoptotic index was defined as the percentage of TUNEL-positive cells among DAPI-positive cells. Data are presented as mean ± SD. One-way ANOVA followed by Tukey's post-hoc test was used for multi-group comparisons (Ctrl vs. POCD and POCD vs. POCD + KN-93). *p < 0.05, **p < 0.01, ***p < 0.001, ****p < 0.0001; ns: no significance.

Western blot analysis demonstrated that, compared with the Ctrl group, the protein levels of CaMKIIδ, p-NF-κB, and Bax were significantly upregulated in the POCD group, whereas the anti-apoptotic protein Bcl-2 was significantly downregulated. Total NF-κB expression remained unchanged, and the p-NF-κB/NF-κB ratio was significantly increased in the POCD group. KN-93 intervention significantly reversed these aberrant protein profiles. The elevated levels of CaMKIIδ, p-NF-κB, and Bax were reduced, and Bcl-2 protein level was restored (Figures 7B,C).

Consistent with these molecular alterations, we further evaluated inflammatory changes in both peripheral circulation and the hippocampus. First, serum concentrations of TNF-α, IL-1β, and IL-6 were detected by ELISA. Compared with the Ctrl group, serum levels of all three pro-inflammatory cytokines were significantly elevated in the POCD group, and KN-93 treatment significantly attenuated this elevation (Figure 7D). We then measured hippocampal cytokine protein levels by ELISA. The protein concentrations of TNF-α, IL-1β, and IL-6 in the hippocampus were significantly higher in the POCD group than in the Ctrl group, whereas KN-93 intervention markedly reduced these cytokine levels relative to the POCD group, demonstrating that CaMKII inhibition alleviates hippocampal neuroinflammatory responses in POCD rats (Figure 7E).

This peripheral inflammatory pattern closely paralleled the hippocampal cytokine changes described above. Spearman correlation analysis of paired samples showed strong positive correlations between serum and hippocampal cytokine levels across all groups (Supplementary Figures S2A–C). Together, these data indicate that hippocampal neuroinflammation occurs alongside systemic inflammatory activation, and that pharmacological inhibition of CaMKII attenuates both in parallel, supporting crosstalk between peripheral and central inflammatory signals.

TUNEL staining showed that the apoptotic index in the hippocampal CA1 region—defined as the percentage of TUNEL-positive cells among DAPI-positive cells—was significantly increased in the POCD group compared with the Ctrl group. The apoptotic index in the POCD + KN-93 group was significantly lower than that in the POCD group (Figures 7F,G). This was consistent with the expression changes of apoptosis-related proteins, further confirming that CaMKII inhibition attenuated surgery-induced hippocampal neuronal apoptosis.

## 4 Discussion

Postoperative cognitive dysfunction (POCD) is one of the most common central nervous system complications in elderly patients following general anesthesia and major surgery. Its main clinical manifestations include decreased attention, impaired executive function, and reduced spatial learning and memory (Vu and Smith, 2022). Previous studies have confirmed that hippocampal neuroinflammation and neuronal apoptosis constitute the core pathological basis of POCD onset and progression (Fricke et al., 2021). However, most studies have focused on intervening in a single inflammatory or apoptotic pathway. This makes it difficult to block the complex postoperative cascade of pathological damage, and clinical translation outcomes have been suboptimal (Kapila et al., 2014). Therefore, identifying upstream regulatory molecules that simultaneously govern inflammatory activation and neuronal apoptosis is critical for elucidating the pathogenesis of POCD and developing effective intervention strategies. In the present study, transcriptomic screening combined with in vivo functional validation was used to investigate the role of CaMKIIδ in surgery-induced cognitive impairment. Our findings indicate that abnormal upregulation of CaMKIIδ is closely associated with the initiation of postoperative hippocampal neuroinflammation, neuronal apoptosis, and ultimately cognitive dysfunction. Specifically, *Camk2d* was significantly upregulated in the hippocampus of aged rats following splenectomy, and its temporal expression profile was consistent with the dynamic changes of neuroinflammatory and apoptotic markers.

WGCNA screening in this study revealed that the gene module highly associated with the cognitive impairment phenotype was significantly enriched in the calcium signaling pathway. Among these genes, CaMKIIδ exhibited markedly elevated expression at postoperative day 3 relative to control animals. Its mRNA expression level was far higher than the baseline level observed in the control group. This upregulation magnitude far exceeded that of other differentially expressed genes in the same pathway, such as Tnnc1 and Adra1b. This result is consistent with previous research on surgery-induced hippocampal calcium overload and disruption of calcium homeostasis (Zhang et al., 2018), but it localizes the downstream effector molecule to a specific calcium/calmodulin-dependent kinase. Previous POCD studies have mostly detected total CaMKII or neuron-enriched CaMKIIα. The latter, as a classic effector molecule of synaptic plasticity and long-term potentiation, reflects synaptic functional damage when its activity is downregulated (Tan et al., 2024). In contrast, this study identified CaMKIIδ as a potential key upstream molecule. This subunit has been well characterized as a core effector molecule of calcium overload-induced apoptosis and inflammation in cardiac tissue. However, its injurious upregulation in the brain, particularly under POCD conditions, has rarely been reported. Recent human genetic studies have shown that CAMK2D missense variants can alter kinase activity and disrupt neurodevelopment (Rigter et al., 2024), suggesting that CaMKIIδ possesses functional dimensions in the brain independent of CaMKIIα. Accordingly, the CaMKIIδ upregulation observed in this study likely represents an injurious calcium signaling branch that operates in parallel with, or even precedes, the downregulation of synaptic CaMKIIα. The dramatic rise in CaMKIIδ expression can be explained by its unique biological characteristics. This kinase exhibits low basal expression in the hippocampus under physiological conditions. As an injury-inducible kinase, its transcription is highly sensitive to stress signals such as calcium overload and oxidative stress. Therefore, it can demonstrate induction magnitudes far exceeding those of structural genes within the acute injury window. In contrast, genes such as Tnnc1 and Adra1b in the same pathway are mostly involved in basal calcium sensing and signal transduction, with limited stress-induced amplitude. This low-basal, high-induction characteristic aligns with the role of CaMKIIδ as a calcium signal cascade amplifier and explains why its upregulation is the most prominent among the differentially expressed genes. From the perspective of upstream triggers, surgical trauma induces systemic inflammation, increases blood-brain barrier permeability, disrupts hippocampal calcium homeostasis, and subsequently activates CaMKIIδ transcription, constituting a reasonable cascade that explains its temporal pattern and magnitude.

Temporal analysis in this study showed that time spent in the target quadrant decreased to approximately 10 s and spontaneous alternation in the Y maze was significantly reduced. Hippocampal CA1 neurons displayed disordered arrangement, cell body shrinkage, nuclear pyknosis, and Nissl body loss. At the molecular level, IL-1β, IL-6, TNF-α, and Bax were upregulated, while Bcl-2 dropped to its lowest level. All of these parameters reached their damage peak on postoperative day 3, perfectly coinciding with the CaMKIIδ expression peak. This result is highly consistent with the time windows reported in previous POCD animal studies. Le et al. (2014) found in a partial hepatectomy POCD model in aged rats that hippocampal dendritic spine loss, microglial activation, and elevations in TNF-α and IL-1β peaked on postoperative days 1–3 (He et al., 2012). Electroacupuncture intervention studies also confirmed that hippocampal neuroinflammation peaked around postoperative day 3 (Feng et al., 2017). Cao et al. (2010) systematically reported that surgical trauma induces hippocampal pro-inflammatory cytokine elevation and cognitive impairment in aged rats, with more severe damage in aged individuals. Chen et al. (2022) further noted that transient postoperative neuroinflammation can lead to long-term downregulation of synaptic NMDARs and persistent spatial memory impairment, with the inflammation peak occurring within several days after surgery. The day 3 peak observed in this study is consistent with these reports, suggesting that this time point reflects the molecular mechanisms of surgical stress, peripheral inflammatory signal transduction, and hippocampal neuroinflammatory cascades.

Molecular experiments in this study suggest that CaMKIIδ may serve as a key signaling branch node, simultaneously regulating postoperative hippocampal neuroinflammation and neuronal apoptosis. In the inflammatory pathway, postoperative hippocampal p-NF-κB activation levels increased in synchrony with CaMKIIδ expression. Pharmacological inhibition of CaMKII with KN-93 significantly suppressed NF-κB phosphorylation and reduced the abnormally high expression of IL-1β, IL-6, and TNF-α. This is consistent with previous reports that CaMKIIδ promotes NF-κB nuclear translocation and initiates pro-inflammatory cytokine transcription (Ling et al., 2013; Willeford et al., 2018). This CaMKII-NF-κB axis has also been independently validated in the brain. Song et al. (2018) found that βCaMKII overexpression in the hippocampal CA1 region can induce neuroinflammation and depressive-like behaviors through COX-2/PGE2 and p38 MAPK/ATF2 pathways, while βCaMKII knockdown reverses these inflammatory biochemical changes. Virijevic et al. (2025) observed coordinated changes in hippocampal NF-κB and CaMKII phosphorylation in chronically stressed rats. Song et al. (2019) reported that assembly of the NR2B-CaMKII-PSD95 signaling module can drive LPS-induced hippocampal neuroinflammation. Ding et al. (2025) recently confirmed in a subarachnoid hemorrhage model that P2X7-CaMKII can drive 5-LOX nuclear translocation and exacerbate microglial dysfunction. In the apoptotic pathway, this study found that inhibition of CaMKIIδ simultaneously blocked postoperative hippocampal inflammatory activation and neuronal apoptosis, disrupting the Bcl-2/Bax balance. This is consistent with previous reports, primarily in cardiac ischemia-reperfusion models, that CaMKIIδ regulates Bcl-2/Bax through both indirect inflammatory activation and direct mitochondrial apoptotic signaling (Zhu et al., 2007). Multiple lines of evidence support CaMKII-mediated neuronal apoptosis. Lim et al. (2020) demonstrated that ethanol-activated CaMKII signaling induces neuronal apoptosis through Drp1-mediated mitochondrial fission and JNK1-dependent NLRP3 inflammasome activation. Lu et al. (2013) found in a neonatal hypoxic-ischemic model that elevated CaMKII activity is associated with p38MAPK, NADPH oxidase-derived ROS, and necrotic/apoptotic cell death, and that KN-93 inhibition of CaMKII can reduce brain injury. Compared with previous intervention strategies targeting single inflammatory factors such as IL-1β and TNF-α (Cibelli et al., 2010; Terrando et al., 2010) or single apoptotic mediators such as Bax and caspase, targeting CaMKIIδ can simultaneously interrupt both pathways at the upstream cascade level.

Other calcium-signaling genes within the MEred module, such as *Atp2b4*, *Tnnc1*, and *Adra1b*, may also contribute to the postoperative phenotype, and their relative contributions will require dissection in future studies. Moreover, the strong positive correlation between serum and hippocampal cytokine levels observed in this study supports the classical peripheral-to-central inflammatory signaling axis, whereby surgical trauma activates systemic immunity that is transmitted to the brain through blood-brain barrier disruption and vagal afferent pathways (Fricke et al., 2021; Li et al., 2024; Yang et al., 2020). Within this systemic inflammatory context, the injury-inducible CaMKIIδ upregulation identified here likely represents a hippocampal signaling effector that translates peripheral inflammatory signals into local neuroinflammatory and apoptotic responses.

CaMKII signaling has been shown to intersect with autophagic flux via mTOR and ULK1 phosphorylation in ischemic and neurodegenerative contexts, and autophagy has been implicated in sevoflurane-induced cognitive dysfunction in aged rats (Zhang et al., 2016; Kong et al., 2020). In the present transcriptomic dataset, however, autophagy-related GO terms and the KEGG autophagy pathway were not significantly enriched among the common DEGs or the MEred module genes, and canonical autophagy markers were not differentially expressed (Supplementary Figures S2, S3). Autophagy therefore does not appear to be a major transcriptionally coordinated component of the response examined here, although post-transcriptional regulation cannot be excluded, and direct biochemical assessment of autophagic flux will be required in future studies.

Pharmacological inhibition of CaMKII with KN-93 improved inflammatory responses, cellular apoptosis, and cognitive function, suggesting that CaMKIIδ may participate in the onset and progression of POCD by regulating neuroinflammatory and apoptotic pathways. As a CaMKII inhibitor, KN-93 intervention effectively improved postoperative spatial learning memory and working memory in rats, significantly increasing time spent in the target quadrant and Y maze spontaneous alternation rate. KN-93 also reduced the hippocampal neuronal apoptosis rate. These results are consistent with neuroprotective reports of CaMKII inhibitors in various brain injury models. Vest et al. (2010) reported that TatCN21 is effective against stroke-related neuronal death by blocking autonomous CaMKII activity. Tilianin reduces cerebral ischemic injury by inhibiting CaMKII-dependent mitochondrial apoptosis and MAPK/NF-κB pathways (Jiang et al., 2019). Griem-Krey et al. (2022) reviewed that direct CaMKII inhibitors such as KN-93 can reduce ischemic neuronal injury.

Notably, CaMKIIδ–NF-κB signaling has been reported to promote neuronal survival in brain ischemia models (Ye et al., 2019; Xu et al., 2016), whereas the present study implicates this axis in neuroinflammatory injury and apoptosis under POCD conditions. Emerging evidence in NF-κB biology supports a context-dependent model in which pathway outcome is determined not by a simple on–off switch, but by subunit composition, post-translational modifications, and the duration of activation. In the POCD model, CaMKIIδ remained significantly upregulated from postoperative day 3through day 7; this sustained elevation, superimposed on the intracellular calcium dyshomeostasis and high oxidative stress inherent to aged rats following major surgery, may drive prolonged NF-κB activation and initiate pro-apoptotic transcriptional programs. Taken together, the injurious role of CaMKIIδ observed in this study likely reflects the unique signaling microenvironment of the aged POCD rat brain.

This study has several limitations. First, we lacked a sevoflurane-only group to distinguish the separate impacts of anesthetic exposure and surgical trauma on hippocampal CaMKIIδ activation and cognitive impairment. Our combined injury model nevertheless recapitulates clinical perioperative scenarios where splenectomy is performed under sevoflurane anesthesia. Existing evidence indicates sevoflurane alone only triggers mild, transient CaMKIIδ upregulation, whereas splenectomy induces severe and sustained hippocampal lesions. Uniform sevoflurane treatment was applied to both POCD and KN-93 groups, and the prominent neuroprotective capacity of KN-93 verifies that CaMKIIδ mediates brain injury induced by combined surgical and anesthetic insults. Second, we adopted the pan-CaMKII inhibitor KN-93 without CaMKIIδ-specific antagonists; this agent suppresses all CaMKII subtypes, so the observed protective phenotypes cannot be exclusively ascribed to CaMKIIδ blockade. Further research utilizing shRNA or CRISPR-based CaMKIIδ knockdown is necessary to define the causal function of CaMKIIδ in POCD. Future experiments will introduce sevoflurane-only and sham-operated groups to clarify the individual pathogenic contributions of anesthesia and surgery.

In addition, this study focused on hippocampus-dependent spatial learning and memory, and swimming speed did not differ among groups in either study, excluding gross motor deficits as a confounder of the cognitive measurements, and PFC-dependent cognitive domains were not assessed. Second, the design lacked sham-operated, vehicle-only, and KN-93-only groups, so the individual contributions of anesthesia, surgical trauma, and splenectomy cannot be parsed; these control groups, together with *Camk2d* gain-of-function and knockdown validation, are planned in future work. Third, intraoperative body temperature, blood pressure, blood gases, and blood glucose were not systematically recorded, although all animals received standardized perioperative care and no perioperative deaths occurred. Finally, the autophagy analysis was limited to the transcriptional level, and dedicated open field, rotarod, and NeuN/Iba1/GFAP immunofluorescence assessments were not performed.

## 5 Conclusion

Using a splenectomy-induced POCD model in aged rats, we identified postoperative day 3 as the critical window for cognitive decline, hippocampal neuroinflammation, and neuronal apoptosis. Transcriptomic profiling and experimental validation revealed that CaMKIIδ is significantly upregulated during this period, paralleling NF-κB activation and the dysregulation of Bax/Bcl-2-mediated apoptotic signaling. Pharmacological inhibition of CaMKII by KN-93 attenuated hippocampal neuroinflammation, reduced neuronal apoptosis, and rescued cognitive impairment. Collectively, these findings suggest that CaMKIIδ contributes to the pathogenesis of POCD by regulating hippocampal neuroinflammation and neuronal apoptosis, and may represent a potential therapeutic target for perioperative cognitive protection. Collectively, these findings identify *Camk2d* upregulation as closely associated with the pathogenesis of POCD, paralleling hippocampal neuroinflammation and neuronal apoptosis, and suggest that CaMKII signaling may represent a potential therapeutic target for perioperative cognitive protection.

## Data Availability

The raw data supporting the conclusions of this article will be made available by the authors, without undue reservation.
