# Supplementary material for: Camk2d upregulation is associated with postoperative cognitive dysfunction in aged rats: pharmacological inhibition of CaMKII attenuates hippocampal neuroinflammation and neuronal apoptosis
Source: Front Aging Neurosci. 2026 Oct 12;18:1975677. doi: 10.3389/fnagi.2026.1975677 (PMC13696443; doi:10.3389/fnagi.2026.1975677)
Supplement: Supplementary file 1 [file Data_Sheet_1.docx]

**Supplementary Figure S1. Correlation between serum and hippocampal cytokine levels in POCD model rats.** (A–C) Correlations between serum and hippocampal levels of TNF-α (A), IL-1β (B), and IL-6 (C) across all paired samples (n = 20; Ctrl, POD3, POD5, and POD7, n = 5 per group). Serum cytokines were measured by ELISA and expressed as pg/mL; hippocampal cytokines were measured by ELISA and normalized to total homogenate protein (pg/mg protein). Solid lines represent linear regression fits with 95% confidence bands (shaded areas). Spearman's rank correlation coefficient (ρ) and P values are shown in each panel. Each dot represents one rat.

**Supplementary Figure S2. Correlation between serum and hippocampal cytokine levels in the KN-93 intervention study.** (A–C) Correlations between serum and hippocampal levels of TNF-α (A), IL-1β (B), and IL-6 (C) in the Ctrl, POCD, and POCD + KN-93 groups (n = 5 rats per group; n = 15 paired samples). Serum and hippocampal cytokine levels were measured by ELISA and expressed as pg/mL. Solid lines represent linear regression fits with 95% confidence bands (shaded areas). Spearman's rank correlation coefficient (ρ) and P values are shown in each panel. Each dot represents one rat.

**Supplementary Figure S3. Over-representation analysis of autophagy-related genes in the common DEGs and the MEred module.** Observed numbers of autophagy-annotated genes (blue bars) are compared with the numbers expected by chance (gray bars), based on the annotated gene proportions in the expressed background (15,729 genes), for the common DEGs shared by all three postoperative time points and for the genes in the MEred module. Two autophagy gene sets were tested: GO autophagy terms (GO:0006914 and all descendants; left panel) and the KEGG autophagy pathway (rno04140; right panel). Fold enrichment (FE) and P values (Fisher's exact test, Benjamini–Hochberg corrected) are shown above the bars. No autophagy gene set was significantly enriched in either gene set (all FE < 1, adjusted P > 0.7).

**Supplementary Figure S4. Individual-level correlation between Camk2d expression and the MEred module eigengene.** Scatter plot of Camk2d expression (log2 size-factor normalized counts, x-axis) against the MEred module eigengene (the first principal component of the standardized expression of module genes, y-axis) across all 12 RNA-seq samples (Ctrl, POD3, POD5, and POD7; n = 3 per group). The solid line represents the linear regression fit. Pearson's correlation coefficient (r = 0.83, P = 7.7 × 10⁻⁴) and Spearman's rank correlation coefficient (ρ = 0.99, P = 4.1 × 10⁻⁹) are shown. Each dot represents one rat.

**Supplementary Figure S5. Correlations of the MEred module eigengene and Camk2d expression with cognitive behavioral parameters at the individual animal level.** Heatmap showing Pearson correlation coefficients (r) between expression measures (MEred module eigengene, the first principal component of the standardized expression of module genes; and Camk2d expression, log2 size-factor normalized counts) and cognitive behavioral parameters (Y-maze spontaneous alternation, time spent in the target quadrant and platform crossings in the MWM probe test) across all 12 RNA-seq samples with paired behavioral data (Ctrl, POD3, POD5, and POD7; n = 3 per group). Pearson's r and P values are shown in each cell; the color scale represents Pearson's r. Spearman's rank correlation coefficients yielded consistent results and are reported in the Results. Each cell represents one correlation across 12 rats.

**Figure 5A**

**Supplementary Figure S6. Western blot images of Figure 5A proteins.** (A) Three independent biological replicate blots of CaMK2δ; (B) Three independent biological replicate blots of Bax.

**Supplementary Figure S7. Western blot images of Figure 5A proteins.** (A) Three independent biological replicate blots of Bcl-2; (B) Three independent biological replicate blots of pNF-κB/NF-κB.

**Figure 7B**

**Supplementary Figure S8. Western blot images of Figure 7B proteins.** (A) Three independent biological replicate blots of CaMK2δ; (B) Three independent biological replicate blots of Bax.

**Supplementary Figure S9. Western blot images of Figure 7B proteins.** (A) Three independent biological replicate blots of Bcl-2; (B) Three independent biological replicate blots of pNF-κB/NF-κB.
