# Supplementary material for: Camk2d upregulation is associated with postoperative cognitive dysfunction in aged rats: pharmacological inhibition of CaMKII attenuates hippocampal neuroinflammation and neuronal apoptosis
Source: Front Aging Neurosci. 2026 Oct 12;18:1975677. doi: 10.3389/fnagi.2026.1975677 (PMC13696443; doi:10.3389/fnagi.2026.1975677)
Supplement: Supplementary file 2 [file Table_1.DOCX]

| Gene | Forward Primer | Reverse Primer |
| --- | --- | --- |
| *Adcy8* | GAGGATGAGGAGGACGAGTTG | GGTGATGGTGGTGTTGATGAG |
| *Grin3* | CAGCAGAACATCCGCAAGTT | GAGGATGAGGATGAGGATGTG |
| *Atp2b4* | TGAAGAAGGTGGTGGATGAG | GGTGGTGATGGTGGTGTAATG |
| *Camk2d* | AAGAAGGTGGAGATGGTGAAG | GATGGTGATGGTGGTGATGTG |
| *Tnnc1* | ATGGCCTGCGTCTTCTTCTG | GGTGGTGATGGTGGTGATGTG |
| *Adra1b* | TCTGCTGGTGGTGATGATGTC | CAGGTGGTGAAGGTGATGAAG |
| *Cckbr* | GCTGGCTGGTGATGATGAAG | GGTGGTGAAGGTGGTGATGTG |
| *Grin2d* | GAAGATGGTGGTGGATGAGTTC | GGTGGTGATGGTGGTGAAGTC |
| *Bax* | TTGCTACAGGGTTTCATCCAG | AGCTCCATGTTGTTGTCCAG |
| *Bcl2* | TGAACCGGCATCTGCACAC | AGAGTCTTCAGAGACAGCCAG |
| *Gapdh* | ACAGCAACAGGGTGGTGGAC | TTTGAGGGTGCAGCGAACTT |

Supplementary Table S1: Primers in this experiment
